# Theoretical modelling of liquid–liquid phase separation: from particle-based to field-based simulation

**DOI:** 10.52601/bpr.2022.210029

**Published:** 2022-04-30

**Authors:** Lin-ge Li, Zhonghuai Hou

**Affiliations:** 1 Hefei National Laboratory for Physical Sciences at the Microscale & Department of Chemical Physics, University of Science and Technology of China, Hefei 230026, China

**Keywords:** Liquid–liquid phase separation, Theoretical modelling, Coarse-grained simulation

## Abstract

Liquid–liquid phase separation (LLPS) has proved to be ubiquitous in living cells, forming membraneless organelles (MLOs) and dynamic condensations essential in physiological processes. However, some underlying mechanisms remain challenging to unravel experimentally, making theoretical modeling an indispensable aspect. Here we present a protocol for understanding LLPS from fundamental physics to detailed modeling procedures. The protocol involves a comprehensive physical picture on selecting suitable theoretical approaches, as well as how and what to interpret and resolve from the results. On the particle-based level, we elaborate on coarse-grained simulation procedures from building up models, identifying crucial interactions to running simulations to obtain phase diagrams and other concerned properties. We also outline field-based theories which give the system's density profile to determine phase diagrams and provide dynamic properties by studying the time evolution of density field, enabling us to characterize LLPS systems with larger time and length scales and to further include other nonequilibrium factors such as chemical reactions.

## INTRODUCTION

Cellular compartmentalization plays an essential role in biological function, from providing spatiotemporal organization of cellular materials to regulating biochemical reactions. It is recently proved that liquid–liquid phase separation (LLPS) is ubiquitous in cells and often underlies the formation of some compartments, either forming organelles that lack delimiting membranes (namely membraneless organelles) such as Cajal bodies and nucleoli or generating dynamic biomolecule condensates facilitating physiological processes (Boeynaems* et al.* 2018; Brangwynne* et al.* 2009, 2015; Hyman* et al.* 2014; Molliex* et al.* 2015). While experiment discoveries are mounting on the significant role LLPS plays in diverse cell functions, some underlying mechanisms remain challenging to determine experimentally, considering the disordered nature of such assemblies. Consequently, it is of great significance for theoretical and computational approaches to assist in unraveling various biophysical phenomena. In this regard, there have been many excellent theoretical works on biological condensation with separate modeling scales and methods aiming at different systems (Alshareedah* et al.* 2020; Bracha* et al.* 2019; Chang* et al.* 2017; Gasior* et al.* 2019; Guillén-Boixet* et al.* 2020; Monahan* et al.* 2017; Nott* et al.* 2015; Wei* et al.* 2020). Nevertheless, a comprehensive physical picture is still required for those who want to get started on a theoretical interpretation.

In this protocol, we aim to present a conceptual framework on selecting suitable methodology and what to interpret and answer from the simulation or theoretical results, which is organized as follows. We briefly introduce the general physical picture of phase separation and how it is applied in biophysical studying. Then we detail the major theories and methodologies required for a simulation or analytical studying, with related examples specifically described and discussed. Finally, we discuss other essential aspects for biomolecular condensates and future research routes.

### A primer on biophysical phase separation

LLPS in cells is a collective process that generally involves multiple components with complex interactions, during which the biomolecules can condense into the formation of a dense phase coexisting with a dilute one. From the perspective of equilibrium statistical physics, such biophysical phenomenon is governed by the change of free energy *F*, which consists of enthalpic and entropic terms:

1 ${\Delta}F={\Delta}{H}-T {\Delta}S. $

**Figure 1:**
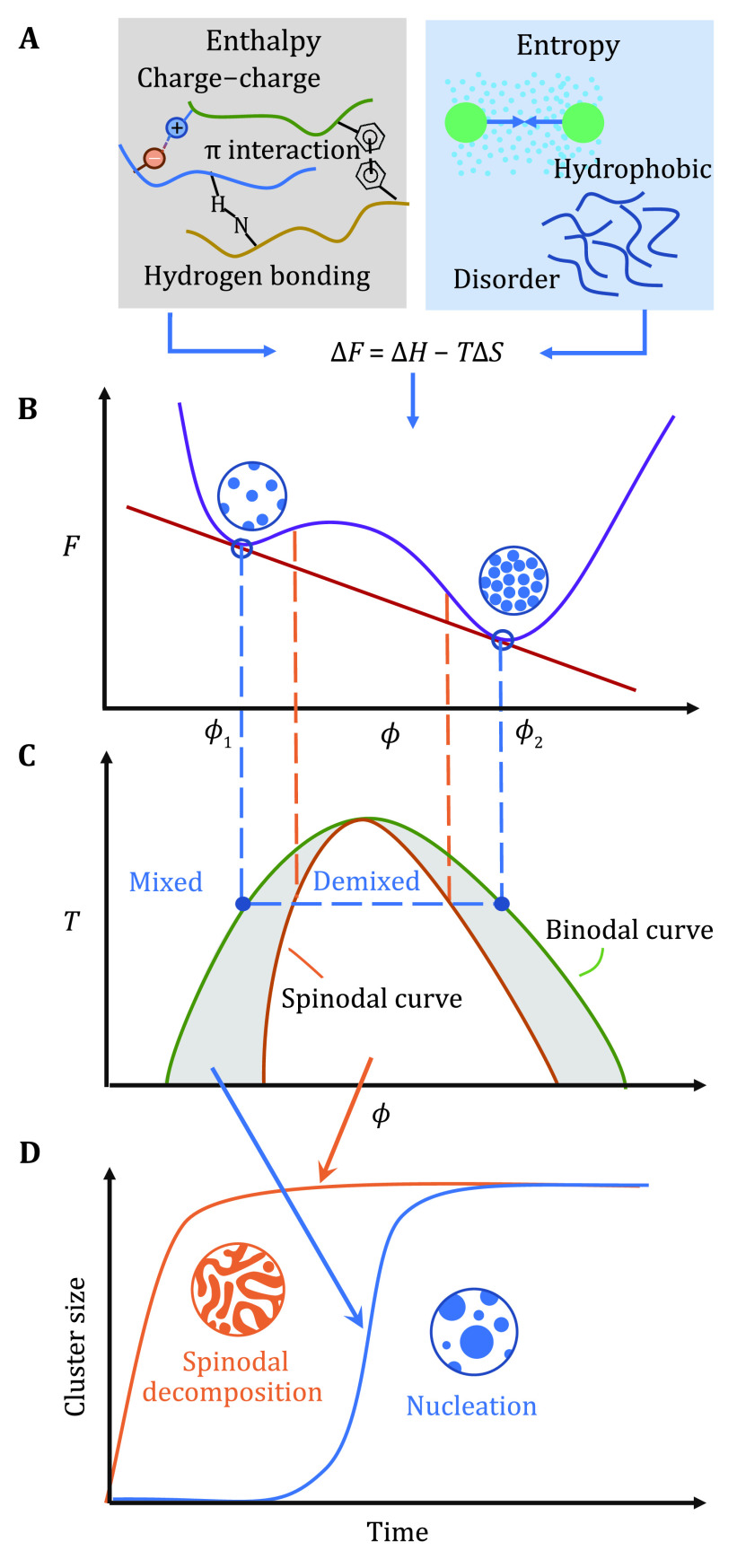
Thermodynamics and dynamics of equilibrium phase separation. **A** Driving forces in LLPS, some enthalpic and entropic effects are listed. **B** Free energy profile. *F* is free energy, *ϕ* is an order parameter such as volume fraction. With two local minima in the free energy curve, the system can phase separate into a dilute phase with volume fraction *ϕ*_1_ and dense phase of *ϕ*_2_. **C** Phase diagram with regions of mixed and demixed phases delineated by the binodal curve. The spinodal curve separates the stable and metastable regions. **D** Two types of dynamic mechanisms on the cluster growth process, namely spinodal decomposition and nucleation

For phase separation to occur, it requires an evident decrease in free energy from mixing to demixing states, *i*.*e*., $ \mathrm{\Delta }{F < 0}_{} $, which corresponds to a negative change in enthalpy ($ \mathrm{\Delta }{H < 0}_{} $) or positive change in entropy ($ \mathrm{\Delta }{S > 0}_{} $) as the driving force. Decreasing enthalpy in biosystem mainly originates from various types of electrostatic interactions, including hydrogen bonds as in between water, proteins and nucleic acids; charge–charge interactions between charged amino acids or nucleotide nucleic acids; and π interactions in which sp^2^ groups or aromatic rings can interact with each other or cations. Regarding entropy, a major aspect is a conformational heterogeneity, as evidence is mounting that many of those phase-separating proteins are disordered or contain intrinsically disordered regions (IDRs) (Burke* et al.* 2015). Another typical example of entropy effect is due to hydrophobic contact ubiquitous in water, in a sense that possible configurations of hydrogen bonding may be restricted at the nonpolar surface of biomacromolecules, which tend to be compacted to minimize the water molecules participating in a solvation shell and maximizes entropic solvation free energy (Huang and Chandler 2002) (Fig. 1A).

Taken together, enthalpy and entropy simultaneously determine the free energy, minimization of which solely governs the phase separation in equilibrium. In a mean-field manner, the free energy may be drawn as a function of an order parameter ($ \phi $) such as volume fraction or concentration, and two phases can emerge when this function is tangent to a single line at two points, *i*.*e*., having two local minima (Fig. 1B). In this context, a system with initial $ \phi $ in between can phase separate into two disparate concentrations $ {\phi }_{1} $ and $ {\phi }_{2} $. Connecting the points of $ {\phi }_{1} $ and $ {\phi }_{2} $ for different temperatures, a *T*–*ϕ* phase diagram can be obtained as shown in Fig. 1C, wherein the green line is known as the binodal curve.

While thermodynamic states give us information on whether or not phase separation does happen, sometimes how it happens, the dynamic mechanism, is of our concern. By finding the points where $ {\partial }^{2}\mathrm{\Delta }{F}^{}/\partial {\phi }_{}^{2}=0 $, a spinodal line can also be drawn in the phase diagram (Fig. 1C, orange line), inside which is the unstable region where phase separation can happen spontaneously and the condensate size surge without induction time (Fig. 1D, orange line) known as spinodal decomposition. However, in the area between spinodal and binodal lines, the system is metastable in which $ \mathrm{\Delta }{F''\left(x\right)}^{} > 0 $, leading to an evident nucleation time required before the rapid growth in condensate (Fig. 1D, blue line). Hence when performing theoretical studying, it is sometimes necessary to pay attention to the dynamic mechanism, especially when compared with experimental observations.

### General framework of selecting theoretical method

In the context of the high degree of complexity in a phase separation system, atomistic simulations using quantum mechanics or atomic molecular dynamics (MD) would usually be severely limited in scope for a given computational budget. Nevertheless, such detailed resolution can be useful in obtaining parameters for larger scales (Murthy* et al.* 2019), capturing inter-residue interaction potentials (Zerze* et al.* 2015) and providing structural characteristics of single protein conformations (Conicella* et al.* 2016). To reduce the level of complexity in particle-based simulation, coarse-graining (CG) is indispensable for constructing practicable protocols, the degree of which can vary from multiple beads per monomer to single bead per molecule (Ruff* et al.* 2019). However, considering the tradeoff between efficiency and accuracy, the choice of coarse-grained resolution should be question-specified, capturing the major physics as well as deploying practical computational resources. Detailed coarse-grained simulation methods will be discussed in the following section.

When investigating phase behaviors, droplet dynamic properties or compartmentalization processes, the coarse-grained simulation that treats each protein individually may again fail in interpreting such large time- and length- scales. From the phenomenological level, one can apply mean-field theories such as the Flory-Huggins theory to obtain constrained free energy profile like Fig. 1B, minimum of which gives density profile of the system to determine phase diagram. To further consider spatial heterogeneity, we can obtain free energy as a function of density $ {\phi \left(r\right)}_{} $. Furthermore, if we take dynamics into consideration, dynamic equations of the time evolution of density field can also be constructed, thus showing us how the phase separation happens. In addition to this framework, we can also involve reaction terms into the change of $ {\phi \left(r\right)}_{} $ to account for frequently encountered chemical reactions in cells. Protocol of field-based theories and simulations will be discussed in detail in the third section.

## SUMMARIZED PROCEDURE

### Select a suitable theoretical method

1　Select a qusestion-specified method. On the basis of accuracy and scale required, choose the particle-based method (Steps 2–8) or the field-based method (Steps 9–12).

### Perform particle-based MD simulation

2　Set up system conditions. Choose proper simulation units, dimensions, boundary conditions and the dynamic equation for MD simulation.

3　Build up a coarse-grained model. Select the residue-based, rigid body or CG particles to describe your molecules.

4　Generate initial configuration of multiple molecules in simulation box to reach target concentration.

5　Identify crucial interactions and parameterization.

6　Run simulations. Warm-up first to avoid extremely unreasonable initial configurations, and then run certain steps until the system reaches equilibration.

7　Proceed simulations under different conditions to obtain phase diagrams. Apply slab method for *T*–*c* phase diagram.

8　Obtain trajectories and analyze simulation results.

### Perform field-based simulation

9　Obtain suitable free energy functional that characterize crucial interactions.

10 Determine unit volume, system size and initialize density profile.

11 Employ dynamic equations for each component and perform numerical simulation on dynamic evolution.

12 Obtain phase diagram, density evolution and other simulation results.

## PARTICLE-BASED SIMULATION

### Molecular dynamics (Step 2)

The general idea of molecular dynamics is to evolve positions of particles according to Newton's equations, which proceeds in the following steps: (1) Set up the initial configuration of model particles; (2) Integrate the particle positions and velocities at each time interval by numerically solving the classical equations of motion:

2 $ {\dot{r}}_{i}={v}_{i},{\dot{v}}_{i}=\frac{{F}_{i}}{{m}_{i}} , $

where $ {r}_{i} $, $ {v}_{i} $, $ {m}_{i} $ and $ {F}_{i} $ are the position, velocity, mass and force vectors corresponding to particle$ i $; (3) Extract the desired properties from the trajectories using statistical mechanics. MD can be performed on a host of software such as LAMMPS (Plimpton 1995), GROMACS (Berendsen* et al.* 1995), and AMBER (Salomon-Ferrer* et al.* 2013).

Although the general scope of MD is quite straightforward, the "particle" in a simulation could vary a lot for different systems. In the context of this LLPS-intended protocol, atomic MD is unpractical compared to an appropriate level of coarse-grained particle method. To describe the motion of such CG particles, one may apply the Langevin equation, which includes a random force term $ \xi \left(t\right) $ generally described by Gaussian white noise to incarnate the role of solvent:

3 $ \,{m}_{i}{\dot{v}}_{i}=-\gamma {\dot{r}}_{i}+{F}_{i}\left(t\right)+\xi_i \left(t\right), $

$ \mathrm{w}\mathrm{h}\mathrm{e}\mathrm{r}\mathrm{e}-\gamma {\dot{r}}_{i} $ is the friction term and $ {F}_{i}\left(t\right) $ is the total deterministic force exerted on particle $ i $ (including interactions and external fields). If the CG particle is of colloid size, as is often the case for biomacromolecules, then it may be described in the overdamped model with its inertial effect ignored, Eq. 3 is reduced to $ \gamma {\dot{r}}_{i}={F}_{i}\left(t\right)+\xi \left(t\right) $. Such an overdamped Langevin equation is mainly applied in our simulations of LLPS.

### Building up a coarse-grained model (Step 3)

**Figure 2:**
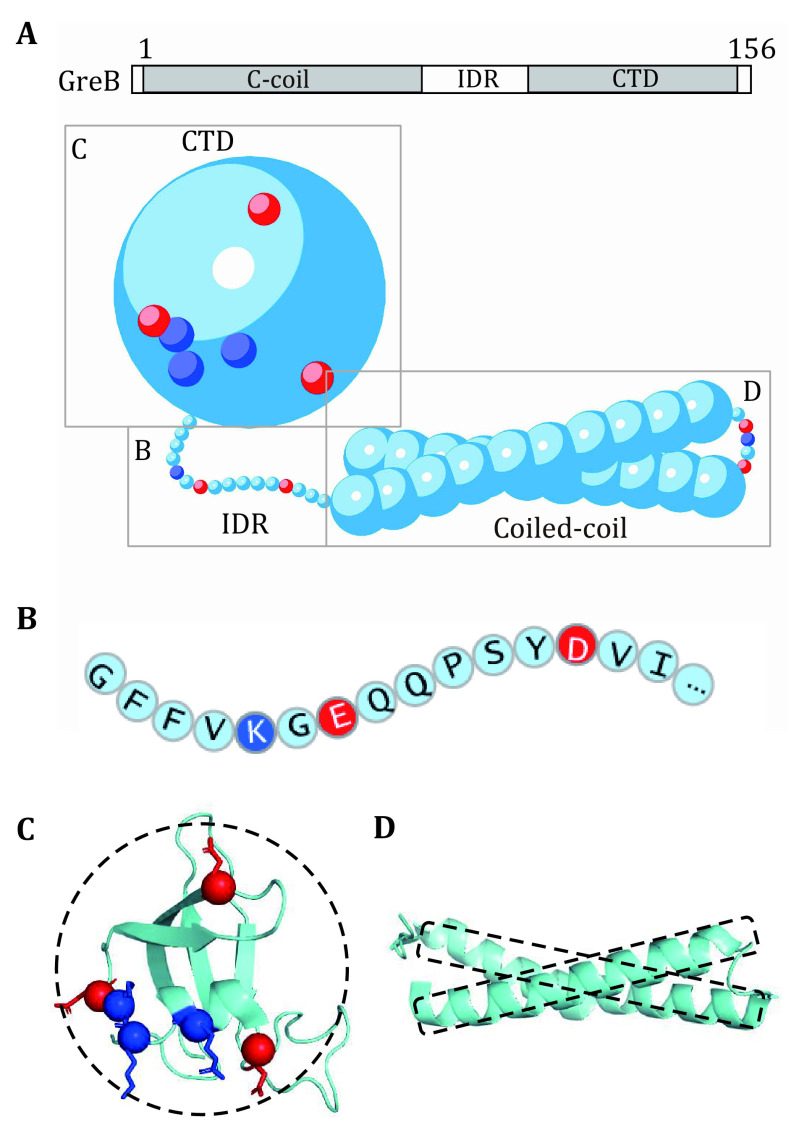
Modeling of proteins with separate levels of coarse grain demonstrated by GreB. **A** Schematic diagram of GreB structural domains and multiscale coarse-grained GreB structure. Detailed coarse-grained methods for each part are given in panels B, C and D. **B** Disordered regions are coarse-grained into residue-based beads, whose charge and interaction parameters are residue specified. In panels C and D, ordered regions are modeled as rigid bodies with coarse geometry according to crystal structures taken from the Protein Data Bank (PDB) and adapted to the model resolution. **C** CTD is modeled as a spherical rigid body with interaction sites. Locations determined from the charged residues (E, D, K and R) on the surface. **D** Coiled-coil region is represented as a dual-rod-like rigid body built with sphere particles outlining the structure for simulation benefit

Coarse-graining means to treat several atoms as a large cluster that updates together. As described earlier, the coarse-grain level may vary from residue-based to the whole macromolecule and should be selected according to the given specified question. In addition, due to the many length- and time- scales involved in protein aggregation, different coarse-grained methods may be taken together to build up a multiscale model. Herein, we take the bacterial transcription factor protein GreB (Borukhov* et al.* 2005) as an example to discuss the following coarse-grain protocol, whose structure contains both disordered and folded regions (Fig. 2A).

The most common degree of coarse-grain for biomacromolecules is to treat each monomer (*e*.*g*., amino acid or nucleotide) in the chain as a single interaction site, classifying particles according to residue types to capture sequence specificity (Dannenhoffer-Lafage and Best 2021; Dignon* et al.* 2018). Since many of the phase-separating proteins are mainly or partially intrinsically disordered, such a residue-based model becomes an appropriate choice, allowing to highlight inter-residue charge effects as well as to reflect the flexibility of the disordered region. Therefore, we chose a residue-based model for disordered IDR of GreB (Fig 2B). Such models are particularly suitable for reproducing sequence-dependent phase behaviors and predicting effects of mutations or PTMs of disordered proteins (Monahan* et al.* 2017).

However, such residue-based models fail to capture the secondary or higher structure of biomacromolecules, hence may no longer be appropriate for situations with folded domains. In this regard, the folded domain can be coarse-grained as a "rigid body" to preserve the structure feature as well as to reduce computation requirements (Dignon* et al.* 2018). As a simple situation, if the domains were near-spherical such as CTD of GreB, it can be approximately regarded as a single large sphere with interaction sites on the surface, also known as the "patchy colloid" model (Bianchi* et al.* 2011; Joseph* et al.* 2021). Radius of the protein sphere can be obtained based on the radius of gyration or corresponding crystal structures taken from the Protein Data Bank (PDB), while locations and strengths of interaction sites can be adapted from crystal structures, NMR or bottom-up computations. Then the sites can be mapped onto the surface of the rigid body according to their relative positions (Fig. 2C, 2D). For other special-shaped structures such as the coiled-coil region in GreB, one can build up a rigid body that captures the geometry outline with a few CG sphere particles (Fig. 2A, 2D). In spite of an approximation, such a model can essentially capture the major physics with high simulation efficiency.

Further coarse-grained models may also be implemented depending on certain time- and length- scales. For example, Guillén-Boixet *et al.* coarse-grained G3BP1 into two beads to capture NTF2 and RRM domain respectively, and RNA is coarse-grained so that each "bead" represents 8–10 nt (Guillén-Boixet* et al.* 2020). Falk *et al.* simulated chromosomes that are constructed from equally sized spherical monomers, with each of them representing 40 kb (Falk* et al.* 2019). Even the most ultra-coarse-grained model treating the entire IDP as a single sphere may be sufficient in specific issues (Bracha* et al.* 2019).

### Identifying crucial interaction (Step 5)

Among many interatomic potentials that exist in a cellular environment, one should identify the essential physical variants responsible for LLPS and ignore the minor ones for simplicity.

#### Exclusive volume

**Figure 3:**
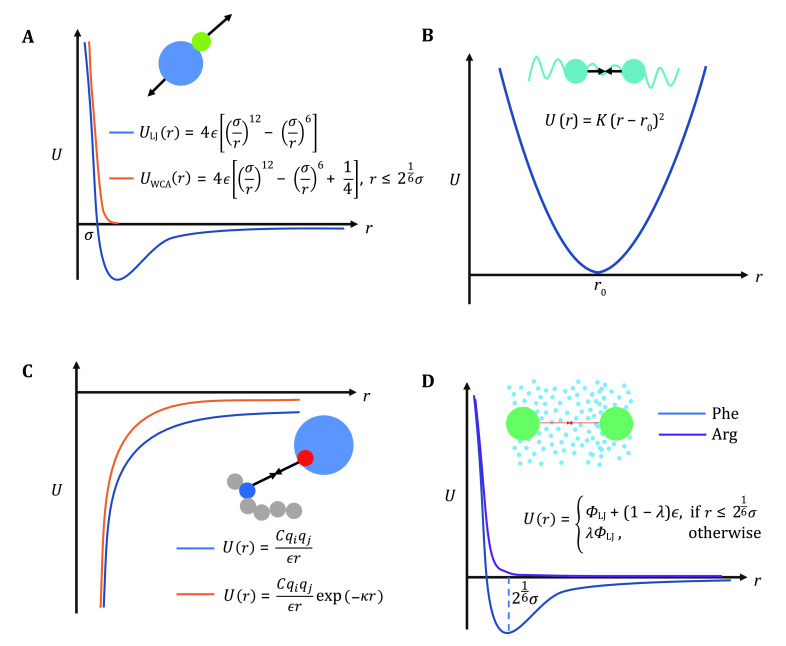
Interaction potentials commonly applied in CG simulation. **A** Exclusive volume with L-J potential (blue line) and WCA potential (orange line). *σ* is the distance when two particles are tangent. **B** Harmonic spring potential for bonded particles, with *K* the spring constant and $ {r}_{0} $ the equilibrium distance. **C** Electrostatic potential with (orange line) or without (blue line) the damping factor “$ \mathrm{exp}\left(-\kappa r\right) $”. **D** Hydrophobic interaction in the HPS model. $ \lambda $ indicates hydrophobicity, $ \lambda =1 $ for Phe and$ \lambda =0 $ for Arg in KR model are drawn as examples

First, we should always consider exclusive volume effects between all pairs of particles, commonly applied via Lennard-Jones (LJ) potential (Fig. 3A, blue line):

4 $ {\mathrm{U}}_{{\rm{LJ}}}\left({r}_{}\right)=4\epsilon \left[{\left(\frac{\sigma }{r}\right)}^{12}-{\left(\frac{\sigma }{r}\right)}^{6}\right]\quad \left(r < {r}_{c}\right),  $

where $ {r}_{c} $ is the cutoff distance, $ \sigma $ denotes the particle diameter, $ {r}_{} $ here is the distance between two coarse-grained beads, the same for other potentials below. Standard 12-6 LJ potential models the joint contributions of steric exclusion and London dispersion. If dispersion is neglected, we can use the Week-Chandler-Anderson (WCA) potential (Fig. 3A, orange line). For steric effects between large domains, softer potentials may be implemented.

#### Bonded interactions

Bonded interactions between connecting neighbor particles can be described by harmonic springs (Fig. 3B):

5 $ {U}_{\text{bond}}\left({r}_{}\right)={K}_{\text{bond}}{\left({r}-{r}_{0}\right)}^{2}/2 , $

where $ {r}_{0} $ is the equilibrium bond length, $ {K}_{\text{bond}} $ is spring constant, magnitude of which can refer to corresponding values derived from interatomic force field (Martin and Siepmann 1998; Mundy* et al.* 1995). Other bonded potentials are also optional such as finite extensible nonlinear elastic (FENE) potential. In addition, a harmonic angular term may also be employed to model the stiffness of chains, such as that of RNAs (Alshareedah* et al.* 2020).

#### Electrostatic interactions

Long-range electrostatic interaction is often a prominent driving force in LLPS, given that RNA is charged and IDPs are commonly enriched in charged amino acids. The basic electrostatic interaction is described in coulomb potential: ${U}_{\mathrm{c}\mathrm{o}\mathrm{u}\mathrm{l}}=\dfrac{C{q}_{i}{q}_{j}}{\epsilon r}$ (Fig. 3C, blue line), as is used in some literatures (Das* et al.* 2018a). In other cases, one could use a Debye−Hückel formalism to reflect the screening effect of polar solvent and ions *in vivo* (Wright 2007) (Fig. 3C, orange line):

6 $ {U}_{\mathrm{c}\mathrm{o}\mathrm{u}\mathrm{l}}\left(r\right)=\frac{C{q}_{i}{q}_{j}}{\epsilon r}\mathrm{exp}\left(-\kappa r\right)\quad \left(r < {r}_{c}\right),  $

and to mimic the screening effect, where $ \epsilon  $ and $ \kappa $ are the dielectric constant and the inverse of Debye screening length, respectively.

#### Hydrophobic interactions

**Figure 4:**
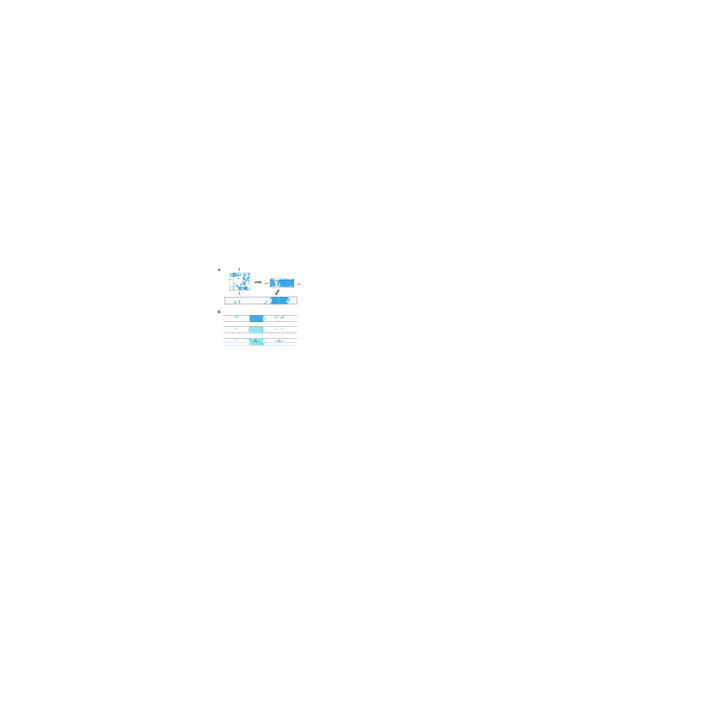
Slab method to generate phase diagram using LAMMPS. **A** Prepare slab-like configuration. Compress the box along *x*- and *y*- dimensions to a length of approximately larger than the size of the macromolecule. Then the system was extended along *z*-dimension. **B** Obtain density profile and phase diagram. Top: slice the simulation box into many pieces (or "chunks") along the *z*-axis, and calculate the particles in each chunk. Middle to bottom: obtain density profile along the *z*-axis, the derivative of which results in two absolute maximum regarded as phase boundary. Calculate the average density in both dilute *ρ*_L_ and dense phases *ρ*_H_

Another commonly asked question is how solvent (usually water) should be modeled and their interactions are handled. The explicit solvent is possible for simulation of a single protein (Wessén* et al.* 2021), but fails at a system size capable of observing LLPS, so that solvent is treated implicitly. That is, solvent-mediated interactions are treated using mean-field descriptions. Apart from the role of screening electrostatic field which can be implicitly embodied into parameterization of $ C $ and $ \kappa $ in the Yukawa potential, hydrophobicity of water can be described with a short-range hydrophobic potential. Applicable models include Kim-Hummer model (Kim and Hummer 2008) or the Hydrophobicity scale (HPS) model, the latter in the form of Eq. 7 (Dignon* et al.* 2018):

7 $ {U}_{{\rm{HPS}}}\left(r\right)=\left\{
\begin{aligned} &
{U}_{\rm{LJ}}+(1-\lambda )\epsilon ,\;\text{i}\text{f}\;\;r\le {2}^{\frac{1}{6}}\sigma \\& \lambda {U}_{\rm{LJ}},\;\qquad\quad\;\;\text{o}\text{t}\text{h}\text{e}\text{r}\text{w}\text{i}\text{s}\text{e}
\end{aligned}\right., $

in which $ {U}_{\rm{LJ}} $ is the standard L-J potential. The λ values, indicating hydropathy, are different for distinct types of residues and can be set referring to literatures (Dannenhoffer-Lafage and Best 2021; Kapcha and Rossky 2014). For example, in Kapcha & Rossky's (KR) HPS (Kapcha and Rossky 2014), Phe residue known with high hydrophobicity has λ = 1 and results in HPS potential similar to L-J potential (Fig. 4D, blue line), while Arg with λ = 0 as a hydrophilic residue has only repulsive volume effect (Fig. 4D, purple line). With the coarse-grained models and these interaction potentials in hand, one can start an MD simulation of LLPS using Eq. 2 or Eq. 3.

### Constructing a phase diagram (Step 7)

A phase diagram can provide powerful mechanism interpretations and insights for experiments. For example, it shows how variables modulate phase separation and whether phase separation can occur in physiologically relevant contexts. By merging phase diagrams of different biosystems, we can also comprehensively interpret how mutations, PTMs, different charge patterns or chemical properties influence phase separation capability.

Generating phase diagrams involves systematically varying two or more state parameters of interest, such as temperature, pressure, pH and salt concentration. For many protein-based systems, the temperature–concentration phase diagram is mainly used, especially for temperature-related proteins such as heat shock factors. The basic procedure is firstly constructing a series of systems with different protein concentrations, and then each system was simulated at a range of temperatures. Trend as a function of a third variable such as mutations can also be drawn in a phase diagram with multiple lines.

One may notice the considerable workload in such a method, with performing an independent simulation in every selected phase point. In particular, at low temperatures where the system evolves slowly to reach equilibrium, such simulation may be time-consuming or even unpractical. As a solution, the slab method is a very efficient strategy and has been successfully implemented in many IDR simulations (Dannenhoffer-Lafage and Best 2021; Kim* et al.* 2010). The basic idea of the slab method includes two main parts. One is to start a simulation with already coexisting dense phase and dilute phase so that molecules can exchange through the interface to reach equilibrium quickly. In the meantime, use the equilibrated configuration in the last temperature as the initial configuration for the current one saves more time. The second is applying slab geometry which is helpful in reducing the system-size effects and reaching high efficiency. Then after a sufficient time for reaching equilibrium, the density of both phases $ {\mathrm{\rho }}_{\mathrm{H}},\;{\rho }_{L} $ can be calculated from the density profile, which determines the phase boundary at the simulation temperature (Fig. 4B). Next, by employing theoretical results from the 3D Ising model, one can obtain a phase diagram through critical condition fitting (Zheng and Mittal 2020).

### Other interpretation of the simulation results (Step 8)

Apart from phase diagrams, many other properties can also be obtained from the simulation results. MD trajectories contain all the information of the simulated system, from microscopic to macroscopic, from statics to dynamics. Thus any desired statistical properties under simulation resolution can be captured. We could calculate thermodynamic properties such as energy and pressure (McCarty* et al.* 2019), or further obtain dynamic information including clusters growth, droplets fusion and mean square displacement (MSD) of molecules (Alshareedah* et al.* 2020). Geometrical aspects can also be statistically quantified, such as the average distance of the chromocenters from the nuclear center (Falk* et al.* 2019) or the density distribution of IDRs along the *x*-axis (Bracha* et al.* 2019). Narrowing down to per-residue insights, one can calculate averaged configuration properties such as averaged protein valence or ratio of intermolecular interaction to intramolecular ones. Such microscopic properties are difficult to obtain experimentally but may provide significant mechanism understandings.

### Approaches alternative to molecular dynamics

Another frequently used simulation algorithm is Monte Carlo (MC). Contrary to MD simulation, MC does not integrate classical equations of motion or evolve the system deterministically, but non-physical moves are performed randomly. In each step, a random move of the system is performed in the conformation space, and whether to accept the move or not depends on acceptance probability based on detailed balance,

8 $ P=\left\{
\begin{aligned} &
{\rm{exp}}(-\beta \Delta E),\;\Delta E > 0\\& 1,\;\qquad\qquad \Delta E\le 0

\end{aligned}\right., $

where $ \beta =1/{k}_{\mathrm{B}}T$, *T* is the simulation temperature, *k*_B_ is the Boltzmann constant, and ∆*E* is the change in energy that accompanies the attempted move. Without force-computing, the whole phase space may be sampled efficiently, with huge speedup in achieving equilibration compared to MD simulations. The MC simulation still has drawbacks, such as the lack of information on physical time scales of processes due to its non-physical moves. Nevertheless, it is an efficient method to investigate thermodynamic phase behaviors (Das* et al.* 2018b). Apart from self-written codes, software such as Material Studio can perform MC simulations. We also refer the reader to LASSI, a lattice-based Monte Carlo engine designed especially for simulations of polymers (Choi* et al.* 2019).

## FIELD-BASED THEORY AND SIMULATION

Compared to particle-based simulations, field-based approaches do not treat each biomolecule individually but describe their collective behaviors from a density field level. Such models enable us to characterize LLPS systems with larger scales, and despite a shortage in describing sequence-specific information, more macroscopic properties and phenomena can be examined qualitatively. Since phase separation is governed by minimization of the global free energy at equilibrium, writing down the free energy function of the system's density profile allows one to construct complete phase diagrams, study dynamic behaviors, analyze thermodynamic properties and parameter dependency in a phenomenological and predictive manner. Therefore, the critical process of field-based modeling is to determine the free energy functional suitable for the given puzzle.

A host of field-based theories has been applied or derived to describe phase separation in biosystems, many of which derived from polymer theories due to the high resemblance of associative polymers and biomacromolecules. A minimum overview of some examples and their implementations in LLPS is provided below.

### Field-based theories (Step 9)

A widely applied model is the Flory–Huggins polymer-solution theory (Flory 1942; Huggins 1942). Considering a homopolymer of length *N* with a volume fraction $ \phi $ in a solvent (with volume fraction $ (1-\phi ) $) on a lattice model, wherein each lattice site is occupied by either a polymer unit or a solvent molecule as a crude account of excluded-volume effects, the Flory–Huggins mixing free energy per site can be obtained under a mean-field assumption:

9 $ \frac{F}{{k}_{\mathrm{B}}T}=\frac{\phi }{N}\mathrm{ln}\phi +\left(1-\phi \right)\mathrm{ln}\left(1-\phi \right)+\chi \phi \left(1-\phi \right) . $

The first and second terms represent the entropy for mixing polymers and solvent molecules, respectively, obtained by counting the possible ways of polymer occupation on the lattice. The third term is the mixing enthalpy, with the Flory parameter $ \chi $ quantifying the interactions as follows:

10 $ \;\chi =\frac{Z}{{k}_{\mathrm{B}}T}\left[{U}_{\mathrm{p}\mathrm{s}}-\frac{\left({U}_{\mathrm{s}\mathrm{s}}+{U}_{\mathrm{p}\mathrm{p}}\right)}{2}\right] , $

where $ {U}_{\mathrm{p}\mathrm{s}} $, $ {U}_{{\rm{ss}}} $ and $ {U}_{\mathrm{p}\mathrm{p}} $ are interaction strengths between polymer-solvent, solvent-solvent and polymer-polymer units. Clearly, a larger $ \chi $ indicates a stronger demixing tendency. Despite a simplified model, the Flory–Huggins theory has been widely used in understanding biological condensates, such as predicting critical points from experimental data (Nott* et al.* 2015), providing explanations for the effects of salts or charge patterns, (Chang* et al.* 2017) inspiring discovery of new interacting components (Gasior* et al.* 2019) and explaining high-dimensional phase behaviors (Falk* et al.* 2019).

To introduce long-range electrostatic effects between oppositely charged polymers, Overbeek-Voorn (O-V) theory extended the Flory–Huggins formalism by applying Debye–Hückel theory for the screened interactions between charges (Overbeek and Voorn 1957) to replace the $ \chi $-related interactions in Eq. 9:

11 $ \frac{F}{{k}_{\mathrm{B}}T}=\frac{\phi }{N}\mathrm{ln}\frac{\phi }{2}+\left(1-\phi \right)\mathrm{ln}\left(1-\phi \right)-\alpha (\sigma \phi {)}^{\frac{3}{2}}, $

where parameter $ \alpha $ is a function of charge per site. With electrostatic interaction taken into account, the O-V theory can be applied in charged biosystems, such as predicting the length dependence of DNA and RNA (Spoelstra* et al.* 2021).

Besides the Flory–Huggins types of mixing entropy and energy, other continuous quantities contributing to the free energy can be taken into account in certain intracellular systems when necessary. For instance, Wei and coworkers (Wei* et al.* 2020) studied liquid–liquid phase separation in an elastic network and proposed a continuum theory with the system's free energy density which reads:

12 \begin{equation*}\begin{split} 
F\left({\phi }_{A}^{d},{\phi }_{A}^{b},\nu ,R\right)=\;&\nu {f}_{\text{mix}}\left({\phi }_{A}^{d}\right)+(1-\nu ){f}_{\text{mix}}\left({\phi }_{A}^{b}\right)\\& +3\nu \frac{\gamma }{R}+\nu {f}_{\text{el}}\left(R\right)\\& -\xi \left[\nu {\phi }_{A}^{d}+\left(1-\nu \right){\phi }_{A}^{b}-{\phi }_{A}^{0}\right],
\end{split}\end{equation*}

wherein the third term denotes the surface tension associated with the phase-separated droplet, the fourth term is the elastic energy related to the network elasticity, and the last term accounts for the conservation of liquid components. They found a scaling relationship between droplet size and shear modulus of the elastic network, which helps to guide fabrications of synthetic cells with desired phase properties.

While the Flory–Huggins theory and its extensions apply site approximation in a mean-field level assumption, one may further consider pair correlations of the interacting residues to reflect sequence features. Accordingly, mean-field theories under pair approximation can also be applied in LLPS such as the Stickers-and-Spacers model. Developed by Semenov & Rubinstein (Semenov and Rubinstein 1998), this model considers the residue groups that participate in attractive interaction as "stickers", and the parts intervening adjacent stickers as "spacers". Based on this architecture, the valence of proteins or other biomolecules can be well-reflected. For illustration, consider a system of macromolecules with $ {n}_{\mathrm{A}} $ and $ {n}_{\mathrm{B}} $ of type A and B interacting with each other with strength $ {\epsilon }_{\mathrm{A}\mathrm{B}} $ , the free energy can be obtained following Semenov & Rubinstein's approach:

13 $ \frac{F}{{k}_{B}T}=-\mathrm{l}\mathrm{n}\mathrm{\Omega }+{N}_{\text{pairs}}\mathrm{ln}\left(\frac{V}{{v}_{b}}\right)-{N}_{\text{pairs}}\frac{\left|{\varepsilon }_{\mathrm{A}\mathrm{B}}\right|}{{k}_{B}T}. $

In the equation, $ \mathrm{\Omega } $ is the combination factor computed from $ \mathrm{\Omega }=\left(\begin{matrix}N{n}_{\mathrm{A}}\\ {N}_{\text{pairs}}\end{matrix}\right)\left(\begin{matrix}N{n}_{\mathrm{B}}\\ {N}_{\text{pairs}}\end{matrix}\right){N}_{\text{pairs}}\text{!}\text{} $, $ {N}_{\text{pairs}} $ is the total number of A–B interact pairs in the system, $ V $ and $ {v}_{b} $ are the system volume and bond volume respectively. As one of its successful applications in LLPS, Wang *et al.* (Wang* et al.* 2018) extended the theory to describe the relationship between the saturation concentration of LLPS and Arg and Tyr residues that are regarded as stickers in FUS family proteins. By minimization of Eq. 13 with respect to $ {n}_{\mathrm{A}} $ and $ {n}_{\mathrm{B}} $, their derivation suggested a straightforward estimation of percolation concentration $ {c}_{\mathrm{p}\mathrm{e}\mathrm{r}\mathrm{c}} $ from the number of stickers:

14 $ {c}_{\mathrm{p}\mathrm{e}\mathrm{r}\mathrm{c}}\sim \frac{1}{{n}_{\mathrm{A}}{n}_{\mathrm{B}}}. $

To describe a biosystem with more accuracy, theories beyond mean-field approximation may also be applied. For instance, random phase approximation (RPA) theory generalized by Lin and Chan was used to describe sequence-specific phase-separation in membraneless organelles (Lin* et al.* 2016). Field-theoretic theories developed for associative polyelectrolyte have also been used to describe biological condensation (McCarty* et al.* 2019).

### Continuum-based simulations (Steps 10–12)

While minimization of the free energy function (or functional) can provide phase properties at equilibrium with the variation of external parameters such as pressure or temperature, it fails at investigating spatial and time evolution to deliver dynamic properties such as spinodal decomposition, fusion/coarsening or compartmentalization processes, which is also of great concern. On the other hand, such processes generally involve quite large spatial or temporal scales, rendering the aforementioned particle-based coarse-grained simulations too expensive and impractical. As a solution, one may perform simulations based on dynamic equations describing the spatial-temporal evolution of the field $ \phi \left(r\right) $ of some relevant order parameters, which could be volume fraction or concentration. Basically, such continuum equations are formulated as partial differential equations derived from basic physical principles such as the conservation of mass or energy. For biological phase separation, dynamic equations that drive the density profile downward the free energy landscape toward thermodynamic equilibrium can be applied, and various phase-transition dynamic models may be applied, such as models A, B, C and H (Hohenberg and Halperin 1977). For example, in Model B, the dynamic equation can be written as:

15 $ \frac{\partial \phi \left(r\right)}{\partial t}=\varGamma {\nabla }^{2}\frac{\delta F\left[\phi \left(r\right)\right]}{\delta \phi \left(r\right)}+{\xi }_{B}\left(r,t\right) , $

where *Γ* is the mobility constant and $ {\xi }_{B} $ is a spatio-temporal noise term characterizing stochastic fluctuations at mesoscopic scales, $F\,[\phi (r)]$ is a free energy function of $\phi (r)$, which locally can take, but not limited to, one of the forms described in the previous section.

Apart from the minimization of free energy as a driving force, other dynamic processes that lead to the change of $ \phi \left(r\right) $ can also be involved in the continuum equations. For instance, chemical reactions are found to bring nonequilibrium features into intracellular phase separation. One example of such reaction-involved continuum simulation is the work on RNA-mediated transcriptional condensates by Henninger *et al.*, in which the protein and RNA dynamics are written as follows (Henninger* et al.* 2021):

16 $ 
\left\{ \begin{aligned} 
&
\frac{\partial {\phi }_{p}}{\partial t}=\frac{{D}_{p}}{{k}_{B}T}\nabla \cdot \left[{\phi }_{p}\left(1-{\phi }_{p}\right)\nabla {f}_{p}\right]\\& \frac{\partial {\phi }_{\rm{RNA}}}{\partial t}=\frac{{D}_{\rm{RNA}}}{{k}_{B}T}\nabla \cdot \left[{\phi }_{\rm{RNA}}\left(1-{\phi }_{\rm{RNA}}\right)\nabla {f}_{\rm{RNA}}\right]\\&\qquad\qquad +{k}_{\mathrm{R}\mathrm{N}\mathrm{A}}{\phi }_{p}-{k}_-{\phi }_{\rm{RNA}}+{D}_{\rm{RNA}}{\nabla }^{2}{\phi }_{\rm{RNA}}

\end{aligned}\right. \;\;\;,$

in which $ {\phi }_{},\;D,\;k $ and $ f $ are concentration, diffusion constant, reaction constant and free energy, and the subscript “p” denotes protein. The first term on the right is diffusion, the second and third terms in RNA dynamics are the production and degradation reaction of RNA. Based on the modeling, they proposed a feedback control mechanism that with the increase of RNA level, the role of RNA changes from promoting condensation to promoting dissolution. As another example, Zwicker* et al*. proposed a continuum description of centrosomes, in which autocatalytic chemical transition between two centrosome material forms was considered to account for two identical centrosomes (Zwicker* et al.* 2014, 2017). Overall, continuum equations allow for efficient simulation of such large systems with long physical time, facilitating systematic investigation of full dynamics of the biological system.

## PERSPECTIVES

**Figure 5:**
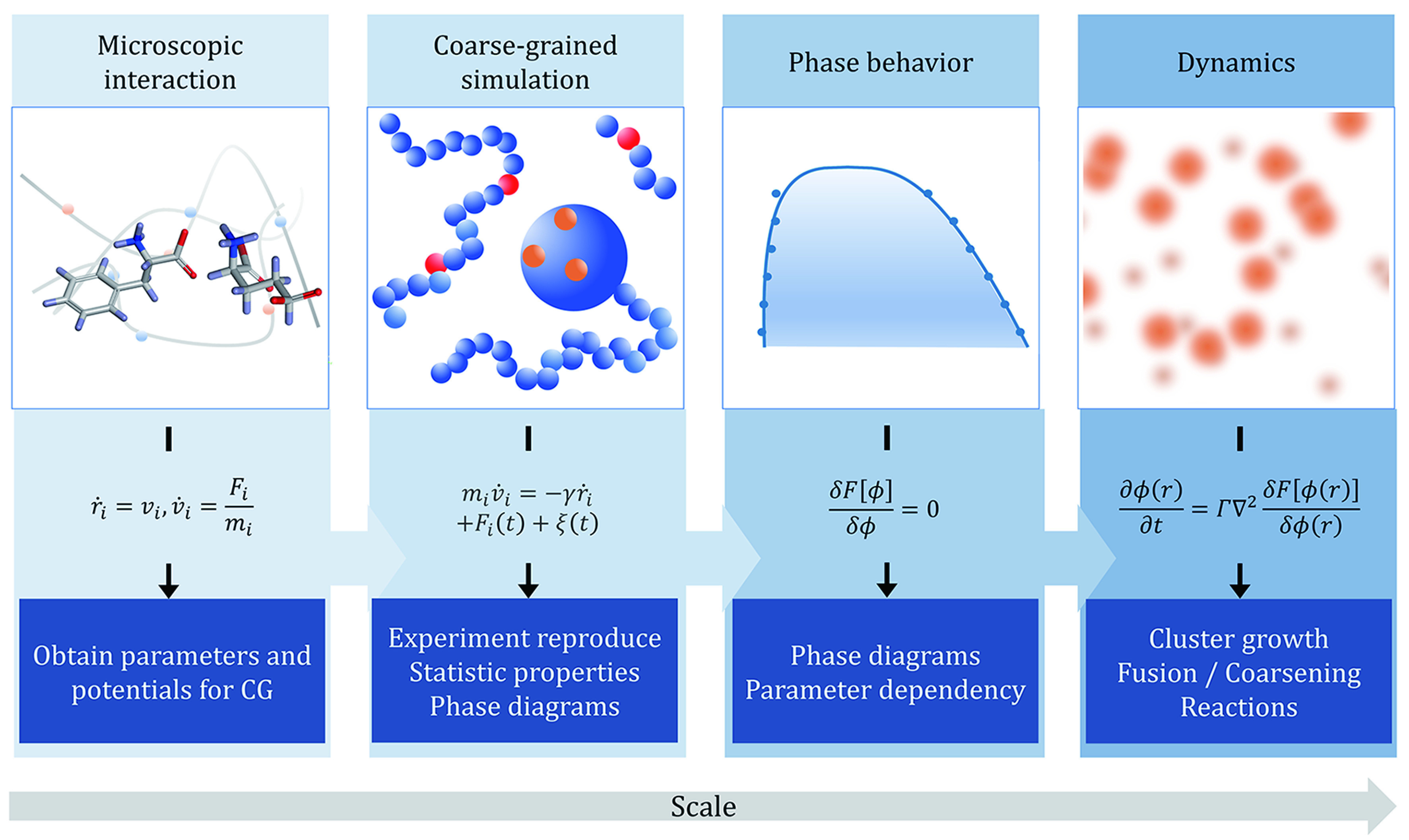
Physical picture of multiscale modeling methods and their applications. From left to right are four levels of modeling method from atomistic level to coarse-grained level and then continuum field scale. The diagrams and equations represent the key methodology in each level, while the bottom boxes highlight the key words of expected results provided by the method

In the protocol, we have introduced methods for unraveling phase separation mysteries in biosystems from a theoretical point of view. Different modeling scales from particle-based to field-based are described, and the global physical picture is summarized as a diagram in Fig. 5. From the atomic level, microscopic interactions and steric effects can be calculated to obtain parameters and potentials for coarse-grained simulations. Then in particle-based simulation, different coarse-grained levels can be applied to reproduce experiments and obtain various statistical properties. Going up to a larger scale, besides high-efficiency simulation strategies, phenomenological theories are introduced to obtain phase diagrams and explore phase behaviors and give parameter dependencies and predictions. Finally, field-based simulations can be used to investigate dynamics in a large time scale. One should recognize that tradeoffs always exist between efficiency and accuracy. While mean-field theories such as Flory−Huggins has good approximation on phase separation behavior, particle-based frameworks will be essential to capture the nuances of sequence-dependent properties.

The given protocol will hopefully enable the readers to get started on a theoretical approach in understanding LLPS. Nevertheless, limitations and challenges still lie ahead. The main challenge is model accuracy and parameter validation. While the current coarse-grain framework can qualitatively understand and reproduce experimentations, quantitative validation and prediction are still difficult. This is due to the mesoscopic nature and complexity of biomolecular condensates, which remains challenging to date for both bottom-up mapping from atomistic computations, and top-down obtaining from experimental measurements. In this regard, the readers looking for quantitatively accurate models are encouraged to obtain parameters from experimental benchmark results, or match from results of various characterization results, such as Hi-C or NMR data (Zhang and Wolynes 2015). The second limitation is to account for nonequilibrium factors *in vivo*. Coarse-grained methods in this protocol are generally oversimplified with only key factors retained, which may well elucidate experiments *in vitro*. However, it should be noted that living-cell environments are much more complicated and are far out of equilibrium. where other nonequilibrium factors, besides aforementioned chemical reactions, may be necessary to be included to explain *in vivo* phase separation. For instance, intracellular species with ATP exerting forces such as filaments may be regarded as active matters, distinguished as a collection of particles converting chemical energy into mechanical work, and has arisen considerable attention in recent years (Jiang and Hou 2014; Needleman and Dogic 2017; Shelley 2016). Active particles have been found to aggregate even in the absence of attracting forces, and novel phase behaviors are found in polymer systems with active matters (Du* et al.* 2019a, b; Gou* et al.* 2021). Introducing such nonequilibrium aspects in the model may help understand some ATP-dependent processes such as the formation of stress granules or nucleoli (Brangwynne* et al.* 2011; Kroschwald* et al.* 2015). Another important aspect of the complex environment *in vivo* would be the crowded cellular medium. Crowding macromolecules have proved to affect the stability and dynamics of protein phase separation significantly (André and Spruijt 2020), and packed medium molecules also introduce evident viscosity to the solution that may highly affect the dynamics of the concerned biomolecules, known as hydrodynamic interactions (Ando and Skolnick 2010). Although beyond the scope of this protocol, we do address these effects as ongoing and future research concerns.

Despite the limitations mentioned above, the current protocol is can already qualitative results for various experimentations. Simulation methods following the protocol can be used to reproduce, understand and eventually predict the effect of the mutation, PTM, change of temperature and other variables. With the appropriate coarse-grained method applied, one can efficiently perform multiple computer simulations with different condition settings, which helps to provide a global picture of parameter space and guide experimentations. In the light of ever-growing computer performance and research developments in both theory and experiments, we foresee an increasingly important role and broad application of theoretical modeling, along with growing capability in understanding physics behind intracellular phase separation.

## Conflict of interest

Lin-ge Li and Zhonghuai Hou declare that they have no conflict of interest.
